# Prognostic significance of dynamic changes in systemic inflammatory markers on mortality after liver transplantation: a retrospective cohort study

**DOI:** 10.7150/ijms.126883

**Published:** 2026-01-14

**Authors:** Eun Jung Kim, Darhae Eum, Jin Ha Park, Seongwook Kang, Jin Sun Cho

**Affiliations:** 1 Department of Anesthesiology and Pain Medicine, Yonsei University College of Medicine, Seoul, Republic of Korea.; 2 Anesthesia and Pain Research Institute, Yonsei University College of Medicine, Seoul, Republic of Korea.

**Keywords:** end-stage liver disease, inflammation, liver transplantation, lymphocyte, neutrophil, postoperative care

## Abstract

**Purpose**: Liver transplantation (LT) is a risky but life-saving treatment for end-stage liver disease. Dynamic changes in systemic inflammation can inform disease progression and postoperative recovery. This retrospective study investigated the prognostic impact of these chronological changes in patients undergoing LT.

**Methods**: Inflammatory statuses were assessed using the neutrophil-to-lymphocyte ratio (NLR), monocyte-to-lymphocyte ratio (MLR), and platelet-to-lymphocyte ratio (PLR) measured preoperatively (within 7 days before surgery) and postoperatively (between days 21 and 90, before any re-exploration). Their predictive performances for three-year postoperative mortality were evaluated. Using the best-performing index, the patients were stratified into normal (persistently low), elevated (low-to-high), normalized (high-to-low), and persistent (persistently high) groups, and associations with mortality were analyzed.

**Results**: A total of 377 patients were included. Among inflammatory indices, the NLR had the highest mortality prediction accuracy. Patients grouped by pre- and postoperative NLR cutoffs (4.2 and 24.0) showed significant mortality differences, with stepwise risk increases from normal to normalized and persistent groups. The NLR-based group was an independent mortality predictor. Compared with the normal group, the normalized and persistent groups had higher mortality, prolonged ventilation, and longer intensive care unit (ICU) and hospital stays.

**Conclusion**: Dynamic changes in systemic inflammation, reflected by pre- and postoperative NLR, were strongly associated with long-term mortality after LT. The NLR is a reliable, accessible inflammatory marker. Elevated preoperative NLR was associated with poor outcomes, with persistent postoperative elevation indicating a worse prognosis than normalization. NLR trajectory may help identify high-risk LT patients and guide postoperative care.

## Introduction

Systemic inflammation contributes significantly to morbidity and mortality in patients with end-stage liver disease. Driven by ongoing liver damage and cirrhosis, inflammation contributes to complications including liver failure, infection, and multiorgan dysfunction, and promotes the development of hepatocellular carcinoma (HCC) 1. In cancer, systemic inflammation typically manifests as a nonspecific reaction to tumor hypoxia, necrosis, or local tissue damage 2. Its clinical significance is emphasized in staging systems and biomarker studies linking it to adverse outcomes in various malignancies, including HCC 3, 4. Systemic inflammation also plays a critical role in both pre- and post-transplantation outcomes in patients undergoing liver transplantation (LT). Pre-transplantation values reflect the severity of liver dysfunction and related complications, thereby influencing surgical risk and survival. Post-transplantation values indicate ongoing inflammation, which contributes to graft rejection, increases susceptibility to infections due to immunosuppressive therapy, and increases the risk of HCC recurrence. Therefore, careful monitoring and management of systemic inflammation throughout the transplantation process are essential to improve prognosis and reduce complications.

Several readily available parameters derived from routine complete blood counts (CBC), such as the neutrophil-to-lymphocyte ratio (NLR) 5, 6, monocyte-to-lymphocyte ratio (MLR) 7, and platelet-to-lymphocyte ratio (PLR) 8, are simple and objective markers of systemic inflammation. These markers have been extensively studied and have been shown to predict patient outcomes 9-11, underscoring their clinical utility 12-14. Their prognostic value is comparable to those of established factors such as multifocality 15, 16, vascular invasion 15, 17, and tumor size 18, 19.

In addition to static values, temporal changes related to surgical progress (e.g., preoperative vs. postoperative) have also gained relevance for outcome prediction 20. Studies investigating time-dependent changes in inflammatory markers have suggested that persistently elevated levels, despite a reduction in disease burden, may reflect ongoing systemic inflammation and predict poor outcomes 3, 21.

We hypothesized that postoperative systemic inflammation in the non-disease-bearing or “neo-organ” state after LT may reflect intrinsic host-related inflammatory status. Accordingly, the normalization of systemic inflammation after surgery may be associated with improved survival. To test this hypothesis, we evaluated the prognostic impact of dynamic changes in CBC-based inflammatory markers in LT recipients, focusing on the transition from high preoperative to low postoperative inflammatory status.

## Patients and Methods

### Patient cohort and ethical considerations

This retrospective cohort study reviewed the medical records of patients who underwent LT at the single center between January 2016 and December 2020. Patients aged < 18 years, who underwent reoperation, and with insufficient data to calculate inflammatory indices were excluded. The study protocol was approved by the Institutional Review Board (IRB no. 4-2025-0099) of Yonsei University Health System. Hospital Research Ethics Committee, which waived the requirement for informed consent because of the retrospective design. This study was conducted in accordance with both the principles of the Declaration of Helsinki, and reported in accordance with the Strengthening the Reporting of Observational Studies in Epidemiology (STROBE) guidelines.

### Data collection and measurement of inflammation-based prognostic markers

Patient data were collected from the hospital electronic medical records. The information retrieved included patients' demographic characteristics and comorbidities, including the etiology of the underlying liver disease, Model for End-stage Liver Disease (MELD) score, and donor type (living/deceased). Preoperative laboratory parameters included hemoglobin value; platelet, white blood cell, and differential counts; estimated glomerular filtration rate; and glucose, creatinine, C-reactive protein, alpha-fetoprotein, and albumin levels. Preoperative laboratory values were defined as the results of the most recent blood tests conducted within one week before surgery. Postoperative laboratory values were obtained from tests performed 21-90 days after surgery before any re-exploration. This period was chosen because postoperative trauma typically resolves and systemic inflammatory responses return to preoperative levels within this timeframe 22, while re-exploration procedures may have affected these responses. When available, values closest to day 21 were selected, with the actual measurements time distribution being 23.4 ± 8.6 days (mean ± SD). Postoperative complications during hospitalization were assessed, including acute kidney injury (as defined by the Kidney Disease: Improving Global Outcomes guidelines) 23, 24, stroke, myocardial infarction, heart failure, prolonged mechanical ventilation (> 48 hours), and re-exploration for hemostasis. Graft failure was evaluated within three years after surgery. Additionally, the length of stay in the intensive care unit (ICU) and hospital, and the one-, two-, and three-year postoperative mortality rates were assessed. Mortality data were obtained from hospital records or the Ministry of Public Administration and Security. Follow-up mortality was analyzed using a time-to-event analysis, with post-transplant survival time defined as the period from the date of transplantation to the date of death.

We assessed inflammatory status preoperatively and postoperatively using NLR, MLR, and PLR. The NLR was calculated as the absolute count of neutrophils (number/μl) divided by the absolute count of lymphocytes (number/μl). The MLR was calculated as the absolute count of monocytes divided by the absolute count of lymphocytes (number/μl). The PLR was calculated as the absolute count of platelets (number/μl) divided by the absolute count of lymphocytes (number/μl). Elevated NLR, MLR, and PLR values were considered to reflect a highly inflammatory state 25, 26.

### Outcome assessment

The primary outcome was three-year postoperative mortality, and our main objective was to evaluate and compare the predictive value of inflammatory indices (NLR, MLR, and PLR) for this outcome. We evaluated the associations between NLR, MLR, and PLR and three-year mortality in all organ recipients. We identified the inflammatory index most strongly associated with mortality and established preoperative and postoperative cutoff values. Based on these cutoff values, we then stratified the patients into four groups and analyzed the survival probabilities for each group. The secondary outcome was the identification of risk factors for mortality.

### Statistical analysis

All statistical analyses were performed using R 4.3.2 (Vienna, Austria; http://www.R-project.org/). *P* < 0.05 was considered statistically significant. Continuous variables were not normally distributed according to the Kolmogorov-Smirnov test; therefore, they were analyzed using the Mann-Whitney U test and presented as medians (interquartile range). Categorical variables were analyzed using chi-square or Fisher's exact tests and presented as absolute numbers (percentage). We assessed the discriminatory ability of the inflammatory index for predicting mortality using Harrell's concordance index (C-index), which estimates the probability of concordance between predicted and observed responses. The C-index was calculated based on the preoperative value of the inflammatory index. Confidence intervals (CIs) and *P*-values for the C-index were calculated using a bootstrapping method with 1000 resamples. We selected the inflammatory index with the highest C-index for further analysis. The preoperative and postoperative cutoff values for the selected inflammatory index were determined using the maximally selected rank statistical method. After classifying the patients into four groups based on the trends of these values, we used Kaplan-Meier survival curves with log-rank test to evaluate the associations between these groups and postoperative mortality. To identify the potential prognostic factors for mortality, we performed univariate and multivariate analyses using the Cox proportional hazards model. Hazard ratios (HRs) and corresponding 95% CIs were calculated.

## Results

**Figure 1:**
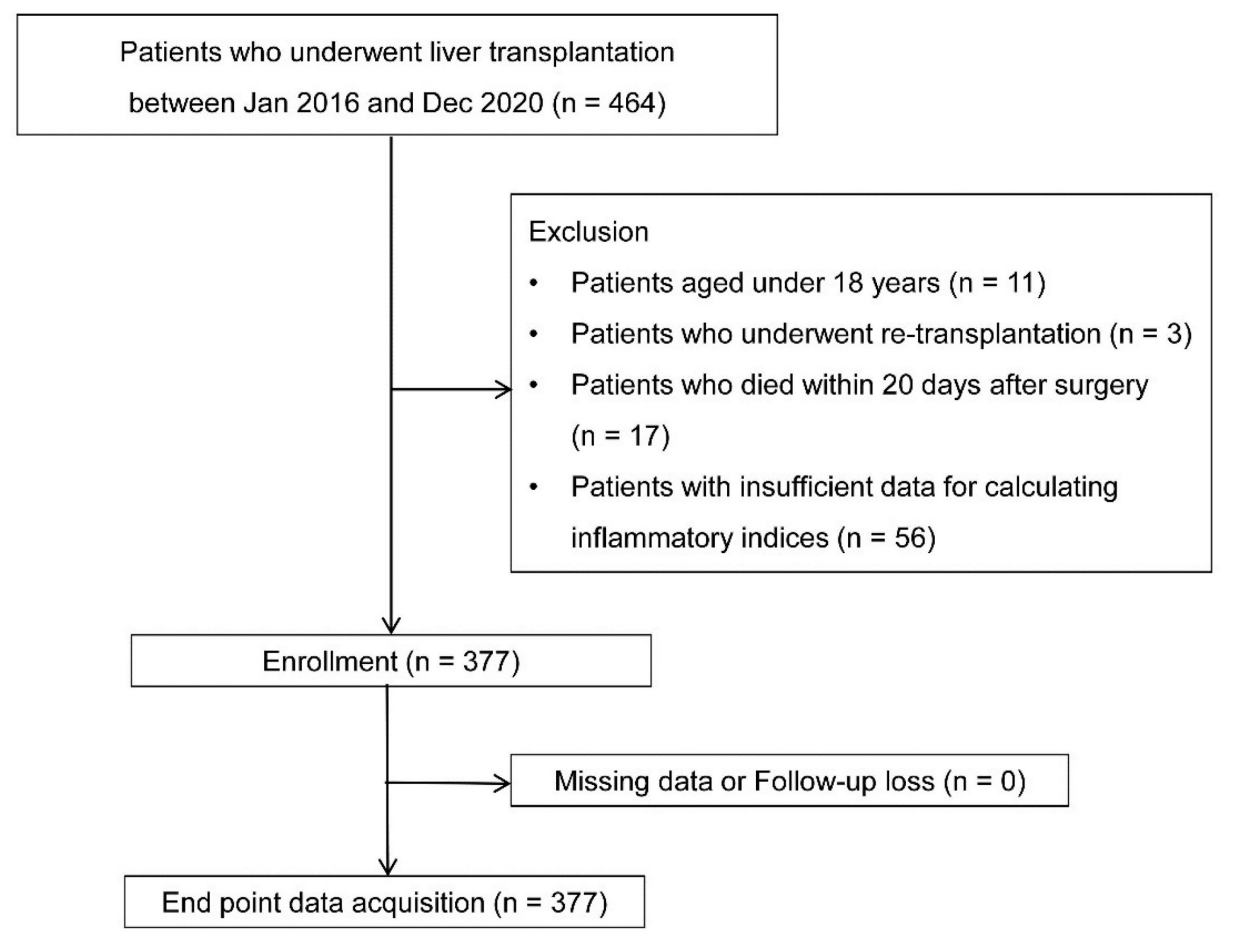
Flow chart.

Among the initial 464 patients, 11, 3, 17, and 56 were excluded due to age < 18, re-transplantation, death within 20 days postoperatively, and insufficient data to calculate inflammatory indices, respectively. The analysis included the remaining 377 patients without missing data or loss to follow-up (Fig. 1).

### Demographics and preoperative laboratory findings

The patient characteristics and laboratory findings are presented in Table 1. Among 377 patients, the three-year postoperative mortality rate was 19.1% (n = 72). Causes of death were infection (n = 51), cancer (n = 9), cardio- or cerebrovascular events (n = 6), and other (n = 6) (Table S1). Deceased patients had a lower BMI (body mass index), higher MELD score, and a higher proportion of preoperative RRT (renal replacement therapy) and transplants from deceased donors than non-deceased patients. Additionally, preoperative hemoglobin levels and platelet counts were lower, whereas C-reactive levels were higher in deceased patients.

Among the inflammatory indices, the preoperative NLR and MLR were higher in the deceased patients, whereas the preoperative PLR did not differ significantly between the two groups. After surgery, the NLR was significantly higher in deceased patients, whereas the MLR and PLR did not differ between the groups (Table 2).

### Prognostic value of inflammatory index for mortality

The prognostic ability of the inflammatory indices for predicting three-year mortality was evaluated using the C-index, which was calculated based on the preoperative values. NLR demonstrated the highest C-index at 0.62 (95% CI 0.55-0.69), which was significantly higher than that of PLR (0.53 [95% CI 0.48-0.60] *P* = 0.039). Although the C-index of NLR was also higher than that of MLR (0.60 [95% CI 0.53-0.66], *P* = 0.195), the difference was not statistically significant.

In addition, in the univariable Cox analysis identifying risk factors for mortality, preoperative NLR (HR 1.02, 95% CI 1.00-1.03, *P* = 0.035) and MLR (HR 1.26, 95% CI 1.03-1.55, *P* = 0.025) were significantly associated with mortality, whereas PLR was not (HR 2.92, 95% CI 0.46-18.54, *P* = 0.256).

While both the C-index and the univariable Cox analysis demonstrated that NLR and MLR were significantly associated with mortality, the NLR demonstrated the modestly higher discriminatory performance and was significantly associated with mortality. Given its performance and collinearity among the indices, the NLR was selected as the primary inflammatory index for further analysis.

### Association of pre- and postoperative NLR-based groups with mortality

To evaluate the impact of preoperative and postoperative NLR on mortality, cutoff values were identified using maximally selected rank statistics: 4.2 for preoperative and 24.0 for postoperative NLR. Based on these, we classified the patients into four groups: Normal (preoperative NLR < 4.2 and postoperative NLR < 24.0; n = 194, 51.2%); Elevated (4.2 and ≥ 24.0; n = 11, 2.9%); Normalized (≥ 4.2 and < 24.0; n = 144, 38.5%); and Persistent (≥ 4.2 and ≥ 24.0; n = 28, 7.4%).

**Figure 2:**
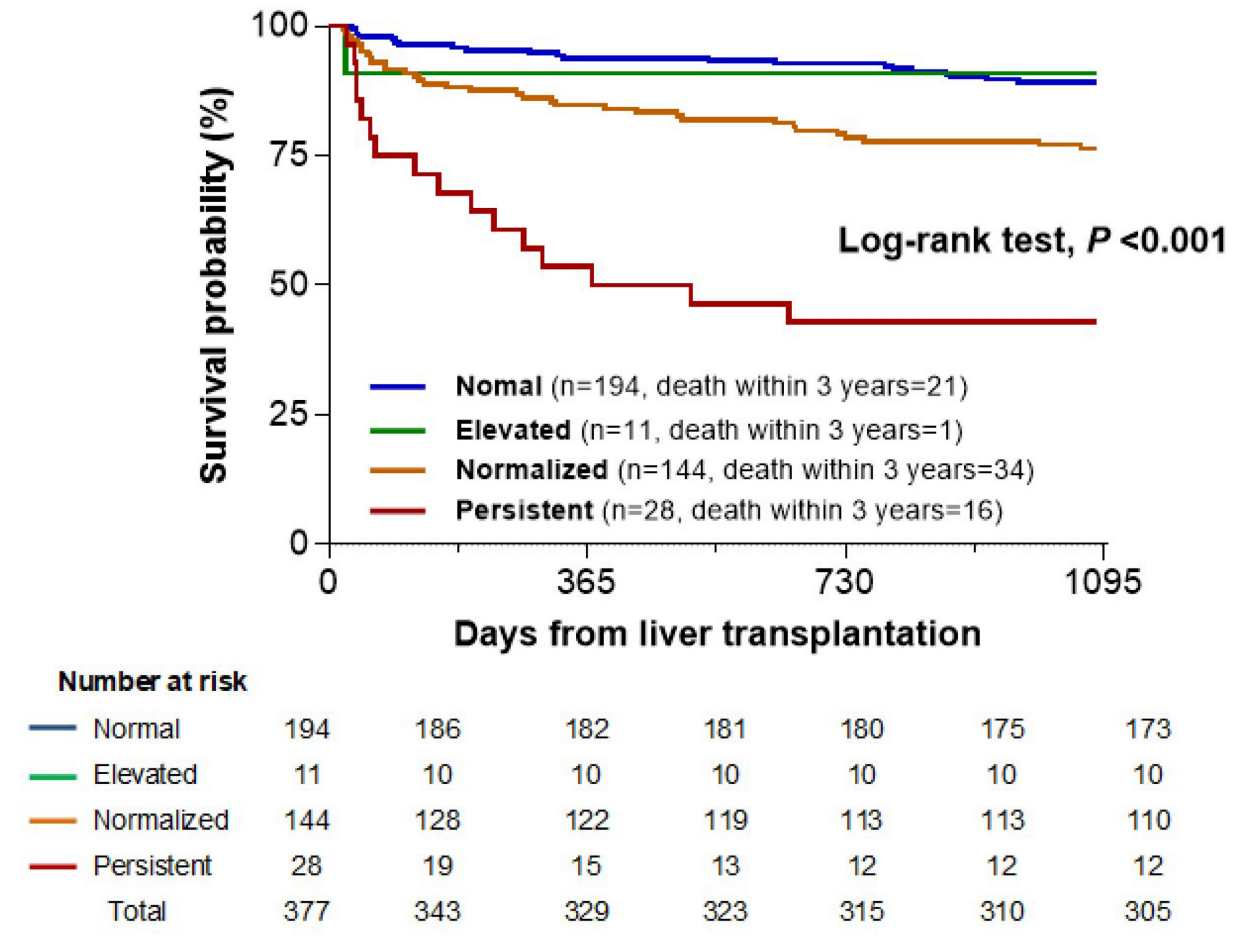
Kaplan-Meier survival curve according to groups stratified by pre- and postoperative neutrophil-to-lymphocyte ratio (NLR) values.

The three-year postoperative mortality rates were 10.8% (21 patients), 9.1% (1 patient), 23.6% (34 patients), and 57.1% (16 patients) in the Normal, Elevated, Normalized, and Persistent groups, respectively. The Kaplan-Meier survival curve showed a significant association between the four groups and three-year mortality (log-rank test, *P* < 0.001). A clear stepwise increase in mortality was observed from the Normal to Normalized groups (log-rank test*, P* = 0.006) and further to the Persistent group (*P* < 0.001). This trend highlighted a graded association, with the Persistent group showing the highest mortality, followed by the Normalized and Normal groups (Fig. 2). However, the Elevated group did not show a significant difference in mortality compared with any other group, possibly owing to the small sample size (*n =* 11) and low number of events (n = 1).

### Risk factors for mortality

The multivariate Cox analysis included the preoperative and postoperative NLR-based group classifications, which were identified as independent risk factors for mortality, along with BMI and platelet count. Compared with the Normal group, the Elevated group had a similar mortality risk, whereas the Normalized (HR 2.22, 95% CI 1.25-3.92, *P* = 0.006) and Persistent groups (HR 6.41, 95% CI 3.16-12.97, *P* < 0.001) had significantly higher risks (Table 3).

### Sensitivity and subgroup analyses

Opportunistic infections occurred in 27 patients (7.2%) between surgery and postoperative NLR measurement: Normal group, 13 [6.7%]; Elevated group, 0; Normalized group, 11 [7.6%]; Persistent group, 3 [10.7%]; *P* = 0.684. Excluding these patients did not alter the significance of the NLR-mortality association in the multivariable Cox analysis (Table S2).

In addition, a subgroup analysis comparing the Normal and Normalized groups was conducted to exclude the influence of underlying disease and assess the prognostic role of inflammation resolution. The Normalized group remained independently associated with higher three-year mortality after adjustment (HR 1.93, 95% CI 1.03-3.62, *P* = 0.040) (Table S3 and S4).

### Postoperative outcomes between pre- and postoperative NLR-based groups

Comparison of postoperative short-term outcomes and one-, two-, and three-year postoperative mortality rates between the preoperative and postoperative NLR groups showed similar characteristics and mortality rates between the Normal and Elevated groups; therefore, these were combined into one group. Compared with the Normal + Elevated group, the Normalized and Persistent groups had significantly higher incidences of prolonged mechanical ventilation (> 48 hours), longer ICU and hospital stays, and higher one-, two-, and three-year postoperative mortalities. The Persistent group also had a significantly higher incidence of heart failure and one-, two-, and three-year postoperative mortality rates compared with the Normalized group. Graft failure rate within three years after surgery did not differ significantly between the groups (Table 4).

## Discussion

In this retrospective cohort study, we evaluated perioperative changes in inflammatory profiles using objective indices and assessed their association with three-year mortality after LT. Among the three inflammatory indices, NLR showed the modestly higher prognostic value and was independently associated with mortality, identifying it as the most reliable marker. Notably, perioperative changes in NLR were significantly associated with mortality. Patients with increased preoperative and postoperative NLR had the highest mortality and morbidity rates, followed by those whose elevated preoperative NLR normalized after surgery. These two groups had worse outcomes than patients with only postoperative elevation or consistently normal NLR.

The main objective of this study was to investigate the prognostic impact of dynamic changes in inflammation-based markers on mortality after LT, addressing the limitations of previous studies focused solely on either pre- or postoperative inflammatory status 7, 25. As elevated levels of inflammatory markers at a single timepoint are associated with worse outcomes but offer limited clinical insights, we focused on temporal changes in inflammation given their relevance to recovery and disease progression.

Dynamic patterns of inflammation have been studied in various conditions, including cardiovascular diseases and cancers, in which inflammation itself contributes to disease progression 20, 22, 27, 28. Moreover, shifts in inflammatory cytokine levels can predict recovery trajectories 27-31. In the present study, we analyzed perioperative changes in the inflammatory status of patients undergoing LT and found that these dynamic patterns were significantly associated with long-term postoperative mortality. Patients were categorized into four groups based on the dynamic changes in inflammatory status before and after LT, which were associated with distinct mortality risks. Patients with normal preoperative marker levels showed favorable outcomes, even if their levels increased postoperatively, similar to those with consistently normal levels. In contrast, patients with elevated preoperative marker levels had significantly higher mortality and morbidity rates, particularly when the levels remained elevated postoperatively. These findings are consistent with those of a previous study in patients with colorectal cancer 20.

Various inflammatory markers, including those from routine blood labs and cytokine-based inflammatory markers are commonly used to evaluate postoperative complications, graft function, rejection, infection, and long-term prognosis 32, 33. Among hematological markers, the NLR has emerged as a reliable and readily available indicator of systemic inflammation and a valuable prognostic tool for solid tumors 34. Neutrophils, the most abundant white blood cells, play key roles in innate immunity, whereas lymphocytes support cellular immunity and help clear malignant cells. Sustained elevation of NLR may reflect a persistent imbalance between systemic inflammation and immune regulation, ongoing subclinical complications, or impaired immune recovery, all of which could contribute to long-term mortality. Given these roles, it is unsurprising that elevated NLR is linked to poor prognosis, tumor recurrence, and increased morbidity and mortality, particularly in patients with HCC after hepatectomy, LT, and radiofrequency ablation 10, 35, 36. NLR can be used to stratify patients according to tumor size, stage, metastatic potential, and lymphatic invasion. It is also an independent prognostic marker for overall, cancer-free, and cancer-specific survival, and is useful for monitoring treatment response.

The MLR has also been explored as a prognostic marker in several cancers and is generally correlated with inflammation and higher mortality 37, 38, consistent with our finding of an association between higher MLR and increased three-year mortality. However, among various inflammatory markers, the most appropriate indicator may vary depending on the patient population. In the present study, although both MLR and NLR were associated with three-year mortality after LT in the univariable Cox analysis, the NLR demonstrated a trend toward better prognostic performance than the MLR. The NLR showed relatively stronger prognostic ability and was identified as an independent risk factor for mortality, suggesting that it may be a more informative marker than the MLR in this specific clinical context.

Another important aspect of this study is its impact on organ transplantation outcomes. In addition to disease control and overall recovery, successful graft integration and functional recovery are critical, particularly given the persistent scarcity of donor organs. This study was designed with these principles in mind and derived clinically meaningful findings. While prior studies have demonstrated associations between inflammatory markers and transplant outcomes, most have focused on preoperative inflammation or static inflammatory levels. In contrast, the present study evaluated the impact of perioperative changes in systemic inflammation on patient outcomes following transplantation. Emerging evidence across diverse clinical settings supports a mechanistic role for dynamic inflammatory changes before and after surgery in disease progression. Notably, in patients with HCC undergoing liver resection outside the transplantation setting, surgical stress-induced inflammation has been shown to promote immune dysregulation, with perioperative changes in the NLR associated with an increased risk of tumor recurrence 39. Consistent with these findings, our findings highlight the potential importance of anti-inflammatory drug interventions in preventing recurrence after LT. While non-steroidal anti-inflammatory drugs, aspirin, and histamine-2-receptor antagonists have been shown to attenuate host-mediated inflammatory responses and may help reduce the risk of cancer recurrence 3, 11, 20, the optimal target population and most appropriate disease type for such interventions remain unclear. Our findings suggest that patients with a high postoperative inflammatory state may be key candidates for such therapies. Nevertheless, other treatment modalities, including chemotherapy, radiotherapy, and immunotherapy, should be considered in conjunction with anti-inflammatory strategies. Furthermore, the potential synergistic effects of combining anti-inflammatory interventions with established treatments merit further investigation.

This study has some limitations. First, owing to the retrospective single-center design, our data were prone to bias; thus, the prognostic significance of the dynamic NLR requires independent confirmation in larger longitudinal multicenter studies. Second, reported NLR thresholds vary across studies, underscoring the need for standardization. The 4.2 threshold identified in a single-center study is informative, but requires validation in broader populations. Nevertheless, as previous studies proposed NLR cutoffs of ≥ 5 10, 36, our finding appears reasonable and consistent with the existing literature. Although the C-index showed acceptable discriminatory ability, it did not reach the level generally considered sufficient for clinical decision-making; therefore, its predictive value should be interpreted with caution. Third, the study cohort comprised patients with HCC of various etiologies, with tumor stages that were not uniform. Therefore, our conclusions require replication within more homogeneous HCC subgroups. Further research is also needed to elucidate the prognostic significance of dynamic changes in inflammation with respect to the patient's HCC type, tumor burden, and characteristics. Fourth, the small sample size in the Elevated (n = 11) and the Persistent (n = 28) groups may limit the reliability of statistical inferences and could lead to inflation of the hazard ratio estimate. Therefore, these results should be interpreted with caution. Lastly, NLR, MLR, and PLR are simple, objective, and cost-effective inflammatory markers derived from routine blood tests, but monitoring changes in pro- and anti-inflammatory cytokines may provide deeper understanding of postoperative inflammatory responses in organ recipients.

In conclusion, our study demonstrated that dynamic changes in inflammatory status, as reflected by preoperative and postoperative NLR, effectively predict three-year mortality in patients undergoing LT. NLR had the highest prognostic value and was independently associated with mortality, indicating that it is the most reliable marker in this population. The measurement of systemic inflammation via the NLR is simple and reliable and can be easily integrated into existing risk stratification strategies. This approach may help clinicians better predict outcomes and guide postoperative management in this high-risk group.

## Supplementary Material

Supplementary tables.

## Figures and Tables

**Table 1:** Demographic, clinical data, and laboratory findings

	All (n = 377)	Non-deceased (n = 305, 80.9%)	Deceased (n = 72, 19.1%)	*P*-value
Age, years	57 (51, 63)	57 (51, 62)	59 (50, 65)	0.304
Sex, male	256 (67.9)	206 (67.5)	50 (69.4)	0.756
BMI, kg/m^2^	23.8 (21.6, 26.1)	24.1 (22.0, 26.7)	22.7 (19.9, 24.1)	< 0.001
Comorbidity				
Hypertension	96 (25.5)	80 (26.2)	16 (22.2)	0.483
Diabetes mellitus	126 (33.4)	99 (32.5)	27 (37.5)	0.415
Chronic kidney disease	24 (6.4)	18 (5.9)	6 (8.3)	0.447
RRT	41 (10.9)	28 (9.2)	13 (18.1)	0.030
MELD score	13.0 (8.6, 23.5)	12.7 (8.5, 21.9)	18.4 (9.4, 32.9)	0.012
Etiology for end-stage liver diseases				
viral/alcoholic	202/124 (53.6/32.9)	161/107 (52.8/35.1)	41/17 (56.9/23.6)	0.525/0.062
biliary/cancer	18/179 (4.8/47.5)	12/139 (3.9/45.6)	6/50 (8.3/55.6)	0.115/0.127
cirrhosis/other	271/43 (71.9/11.4)	223/32 (73.1/10.5)	48/11 (66.7/15.3)	0.274/0.251
Deceased donor	75 (19.9)	53 (17.4)	22 (30.6)	0.012
hemoglobin, g/dl	10.2 (8.6, 12.0)	10.4 (8.8, 12.2)	9.6 (8.1, 11.1)	0.006
platelet count, 10^3^/µl	72 (53, 107)	76 (53, 109)	60 (45, 96)	0.049
glucose, mg/dl	110 (92, 145)	110 (92, 145)	111 (95, 146)	0.625
creatinine, mg/dl	0.78 (0.61, 1.09)	0.76 (0.60, 1.02)	0.86 (0.64, 1.31)	0.051
eGFR	94.4 (62.8, 126.9)	96.6 (67.5, 128.0)	81.6 (49.6, 115.5)	0.041
C-reactive protein, mg/L	4.0 (1.4, 14.0)	3.5 (1.3, 12.8)	6.4 (2.2, 17.0)	0.025
alpha-fetoprotein, ng/dl	4.2 (2.5, 10.1)	4.1 (2.5, 9.2)	6.0 (2.3, 20.5)	0.445
albumin, g/dl	3.1 (2.8, 3.5)	3.1 (2.8, 3.6)	3.2 (2.8, 3.5)	0.958

Values are median (interquartile range) or number (percent). BMI: body-mass index; RRT: renal-replacement therapy; MELD: Model for End-stage Liver Disease; eGFR, estimated glomerular filtration rate.

**Table 2:** Preoperative and postoperative inflammatory indices

	All (n=377)	Non-deceased (n = 305, 80.9%)	Deceased (n = 72, 19.1%)	*P*-value
Preoperative				
white blood cell (/μl)	4190 (2650, 6320)	4120 (2650,6130)	4390 (2590, 7120)	0.482
neutrophil (/μl)	2810 (1603, 4580)	2700 (1620, 4540)	3080 (1690, 5540)	0.225
lymphocyte (/μl)	700 (450, 1040)	720 (480, 1080)	520 (330, 880)	0.001
monocyte (/μl)	320 (210, 480)	320 (210, 480)	300 (210, 470)	0.745
platelet (10^3^/μl)	72 (53, 107)	76 (53, 109)	60 (45, 96)	0.049
NLR	3.83 (2.25, 8.15)	3.55 (2.14, 7.29)	5.79 (3.08, 11.11)	< 0.001
MLR	0.46 (0.28, 0.80)	0.44 (0.27, 0.74)	0.57 (0.31, 1.11)	0.011
PLR	0.11 (0.07, 0.16)	0.11 (0.07, 0.15)	0.13 (0.08, 0.20)	0.080
Postoperative				
white blood cell (/μl)	4980 (3650, 7010)	4950 (3670, 6970)	5280 (3500, 7530)	0.784
neutrophil (/μl)	3850 (2640, 5450)	3840 (2670, 5280)	4010 (2440, 6430)	0.522
lymphocyte (/μl)	510 (340, 740)	530 (360, 770)	400 (240, 590)	< 0.001
monocyte (/μl)	330 (230, 480)	340 (240, 480)	330 (200, 480)	0.155
platelet (10^3^/μl)	148 (102, 203)	154 (112, 209)	102 (61, 184)	< 0.001
NLR	7.51 (4.53, 13.64)	7.09 (4.42, 12.06)	10.56 (4.69, 22.36)	0.009
MLR	0.62 (0.44, 1.03)	0.61 (0.43, 0.96)	0.71 (0.48, 1.30)	0.094
PLR	0.30 (0.19, 0.47)	0.30 (0.19, 0.46)	0.27 (0.16, 0.51)	0.608

Values are median (interquartile range). NLR: neutrophil-to-lymphocyte ratio; MLR: monocyte-to-lymphocyte ratio; PLR: platelet-to-lymphocyte ratio.

**Table 3:** Cox proportional hazard model of mortality after liver transplantation

	Univariate analysis			Multivariate analysis		
	HR	95% CI	*P*-value	HR	95% CI	*P*-value
BMI, kg/m2	0.87	0.81-0.93	<0.001	0.90	0.84-0.97	0.005
RRT	2.05	1.13-3.74	0.019			
MELD score	1.03	1.01-1.05	0.001			
deceased donor	1.94	1.15-3.25	0.013			
hemoglobin, g/dl	0.86	0.77-0.96	0.007			
platelet count, 103/µl	0.995	0.99-1.00	0.061	0.99	0.99-1.00	0.042
C-reactive protein, mg/L	1.01	1.00-1.02	0.033			
Pre-and postoperative NLR-based group						
Normal	Ref			Ref		
Elevated	0.88	0.12-6.53	0.899	0.78	0.10-5.83	0.808
Normalized	2.37	1.38-4.09	0.002	2.22	1.25-3.92	0.006
Persistent	7.79	4.06-14.96	<0.001	6.41	3.16-12.97	<0.001

BMI: body-mass index; RRT: renal-replacement therapy; MELD: Model for End-stage Liver Disease; NLR: neutrophil-to-lymphocyte ratio.

**Table 4:** Postoperative outcomes according to preoperative and postoperative NLR

	Normal + elevated (n = 205, 54.4%)	Normalized (n = 144, 38.2%)	Persistent (n = 28, 7.4%)	*P*-value
acute kidney injury	76 (37.1)	84 (58.3)^*^	17 (60.7)	< 0.001
stroke	0	1 (0.7)	0	0.444
myocardial infarction	2 (1.0)	1 (0.7)	1 (3.6)	0.391
heart failure	0	0	2 (7.1)^*,†^	< 0.001
ventilator care >48h	27 (13.2)	49 (34.0)^*^	11 (39.3)^*^	< 0.001
hemostatic reoperation	11 (5.4)	10 (6.9)	3 (10.7)	0.519
ICU stay, day	4.0 (3.0, 4.0)	5.0 (4.0, 8.0)^*^	4.5 (3.3, 9.0)^*^	< 0.001
hospital stay, day	24.0 (21.0, 34.0)	31.5 (22.3, 46.5)^*^	38.0 (25.3, 61.8)^*^	< 0.001
1-year mortality	13 (6.3)	22 (15.3)^*^	13 (46.4)^*,†^	< 0.001
2-year mortality	15 (7.3)	31 (21.5)^*^	16 (57.1)^*,†^	< 0.001
3-year mortality	22 (10.7)	34 (23.6)^*^	16 (57.1)^*,†^	< 0.001
graft failure within 3 years	8 (3.9)	6 (4.2)	1 (3.6)	0.986

Values are number of patients (%) or median (interquartile range). ^*^, *P* <0.05 compared to the normal + elevated groups; ^†^, *P* <0.05 compared to the normalized group. NLR: neutrophil-to-lymphocyte ratio; ICU: intensive care unit.

## Abbreviations

LT: liver transplantation
NLR: neutrophil-to-lymphocyte ratio
MLR: monocyte-to-lymphocyte ratio
PLR: platelet-to-lymphocyte ratio
HCC: hepatocellular carcinoma
CBC: complete blood counts
IRB: Institutional Review Board
STROBE: Strengthening the Reporting of Observational Studies in Epidemiology
BMI: body mass index
RRT: renal replacement therapy
MELD: Model for End-stage Liver Disease
ICU: intensive care unit
CI: Confidence intervals
HR: Hazard ratios
eGFR: estimated glomerular filtration rate

## References

[1] Dirchwolf M, Ruf AE (2015). Role of systemic inflammation in cirrhosis: From pathogenesis to prognosis. World J Hepatol.

[2] Roxburgh CS, McMillan DC (2010). Role of systemic inflammatory response in predicting survival in patients with primary operable cancer. Future Oncol.

[3] Diakos CI, Charles KA, McMillan DC, Clarke SJ (2014). Cancer-related inflammation and treatment effectiveness. Lancet Oncology.

[4] Liu PH, Hsu CY, Hsia CY, Lee YH, Su CW, Huang YH (2016). Prognosis of hepatocellular carcinoma: Assessment of eleven staging systems. J Hepatol.

[5] Walsh SR, Cook EJ, Goulder F, Justin TA, Keeling NJ (2005). Neutrophil-lymphocyte ratio as a prognostic factor in colorectal cancer. J Surg Oncol.

[6] Thang NVV, Luyen LT, Vi NTT, Hai PD (2025). Neutrophil-to-lymphocyte-to-albumin ratio as a prognostic marker for mortality in sepsis and septic shock in Vietnam. Acute Crit Care.

[7] Mano Y, Yoshizumi T, Yugawa K, Ohira M, Motomura T, Toshima T (2018). Lymphocyte-to-Monocyte Ratio Is a Predictor of Survival After Liver Transplantation for Hepatocellular Carcinoma. Liver Transpl.

[8] Smith RA, Bosonnet L, Raraty M, Sutton R, Neoptolemos JP, Campbell F (2009). Preoperative platelet-lymphocyte ratio is an independent significant prognostic marker in resected pancreatic ductal adenocarcinoma. Am J Surg.

[9] Coussens LM, Werb Z (2002). Inflammation and cancer. Nature.

[10] Gomez D, Farid S, Malik HZ, Young AL, Toogood GJ, Lodge JP (2008). Preoperative neutrophil-to-lymphocyte ratio as a prognostic predictor after curative resection for hepatocellular carcinoma. World J Surg.

[11] Crusz SM, Balkwill FR (2015). Inflammation and cancer: advances and new agents. Nat Rev Clin Oncol.

[12] Gabay C, Kushner I (1999). Acute-phase proteins and other systemic responses to inflammation. N Engl J Med.

[13] Arefhosseini S, Aghajani T, Tutunchi H, Ebrahimi-Mameghani M (2024). Association of systemic inflammatory indices with anthropometric measures, metabolic factors, and liver function in non-alcoholic fatty liver disease. Sci Rep.

[14] Pomacu MM, Trașcă MD, Pădureanu V, Bugă AM, Andrei AM, Stănciulescu EC (2021). Interrelation of inflammation and oxidative stress in liver cirrhosis. Exp Ther Med.

[15] Grazi GL, Cescon M, Ravaioli M, Ercolani G, Gardini A, Del Gaudio M (2003). Liver resection for hepatocellular carcinoma in cirrhotics and noncirrhotics. Evaluation of clinicopathologic features and comparison of risk factors for long-term survival and tumour recurrence in a single centre. Aliment Pharmacol Ther.

[16] Llovet JM, Fuster J, Bruix J (1999). Intention-to-treat analysis of surgical treatment for early hepatocellular carcinoma: resection versus transplantation. Hepatology.

[17] Nagasue N, Ono T, Yamanoi A, Kohno H, El-Assal ON, Taniura H (2001). Prognostic factors and survival after hepatic resection for hepatocellular carcinoma without cirrhosis. Br J Surg.

[18] Yeh CN, Chen MF, Lee WC, Jeng LB (2002). Prognostic factors of hepatic resection for hepatocellular carcinoma with cirrhosis: univariate and multivariate analysis. J Surg Oncol.

[19] Regimbeau JM, Abdalla EK, Vauthey JN, Lauwers GY, Durand F, Nagorney DM (2004). Risk factors for early death due to recurrence after liver resection for hepatocellular carcinoma: results of a multicenter study. J Surg Oncol.

[20] Yasui K, Shida D, Nakamura Y, Ahiko Y, Tsukamoto S, Kanemitsu Y (2021). Postoperative, but not preoperative, inflammation-based prognostic markers are prognostic factors in stage III colorectal cancer patients. Br J Cancer.

[21] Mantovani A, Allavena P, Sica A, Balkwill F (2008). Cancer-related inflammation. Nature.

[22] Chan JCY, Diakos CI, Chan DLH, Engel A, Pavlakis N, Gill A (2018). A Longitudinal Investigation of Inflammatory Markers in Colorectal Cancer Patients Perioperatively Demonstrates Benefit in Serial Remeasurement. Ann Surg.

[23] Section 1 (2012). Introduction and Methodology. Kidney International Supplements.

[24] Cho JS, Shim JK, Lee S, Song JW, Choi N, Lee S (2021). Chronic progression of cardiac surgery associated acute kidney injury: Intermediary role of acute kidney disease. J Thorac Cardiovasc Surg.

[25] Cui S, Cao S, Chen Q, He Q, Lang R (2023). Preoperative systemic inflammatory response index predicts the prognosis of patients with hepatocellular carcinoma after liver transplantation. Front Immunol.

[26] Cho JS, Cho YJ, Shim JK, Jeon Y, Lee S, Choi HW (2024). Risk stratification model integrating nutritional and inflammatory factors for predicting 1-year mortality after valvular heart surgery: a retrospective cohort study. Int J Surg.

[27] Wu M, Yang S, Feng X, Li C, Liu X, Zhang Z (2021). Combining Preoperative and Postoperative Inflammatory Indicators Can Better Predict the Recurrence of Hepatocellular Carcinoma After Partial Hepatectomy. J Inflamm Res.

[28] Li Z, Zhao R, Cui Y, Zhou Y, Wu X (2018). The dynamic change of neutrophil to lymphocyte ratio can predict clinical outcome in stage I-III colon cancer. Sci Rep.

[29] Aly M, Abdalla RN, Batra A, Shaibani A, Hurley MC, Jahromi BS (2021). Follow-up neutrophil-lymphocyte ratio after stroke thrombectomy is an independent biomarker of clinical outcome. J Neurointerv Surg.

[30] Li C, Zhang F, Shen Y, Xu R, Chen Z, Dai Y (2017). Impact of Neutrophil to Lymphocyte Ratio (NLR) Index and Its Periprocedural Change (NLR(Δ)) for Percutaneous Coronary Intervention in Patients with Chronic Total Occlusion. Angiology.

[31] Lin JX, Huang YQ, Wang ZK, Xie JW, Wang JB, Lu J (2021). Prognostic importance of dynamic changes in systemic inflammatory markers for patients with gastric cancer. J Surg Oncol.

[32] Lee DD, Singh A, Burns JM, Perry DK, Nguyen JH, Taner CB (2014). Early allograft dysfunction in liver transplantation with donation after cardiac death donors results in inferior survival. Liver Transpl.

[33] Murakami S, Uchida T, Imamura M, Suehiro Y, Namba M, Fujii Y (2023). Correlation between serum pro-inflammatory cytokine levels and the prognosis of the patients with acute liver failure. J Gastroenterol Hepatol.

[34] Cescon M, Bertuzzo VR, Ercolani G, Ravaioli M, Odaldi F, Pinna AD (2013). Liver transplantation for hepatocellular carcinoma: role of inflammatory and immunological state on recurrence and prognosis. World J Gastroenterol.

[35] Bertuccio P, Turati F, Carioli G, Rodriguez T, La Vecchia C, Malvezzi M (2017). Global trends and predictions in hepatocellular carcinoma mortality. J Hepatol.

[36] Halazun KJ, Hardy MA, Rana AA, Woodland DCt, Luyten EJ, Mahadev S (2009). Negative impact of neutrophil-lymphocyte ratio on outcome after liver transplantation for hepatocellular carcinoma. Ann Surg.

[37] Wang Q, Qiao W, Liu B, Li J, Yuan C, Long J (2022). The monocyte to lymphocyte ratio not only at baseline but also at relapse predicts poor outcomes in patients with hepatocellular carcinoma receiving locoregional therapy. BMC Gastroenterol.

[38] Choi YH, Lee JW, Lee SH, Choi JH, Kang J, Lee BS (2019). A High Monocyte-to-Lymphocyte Ratio Predicts Poor Prognosis in Patients with Advanced Gallbladder Cancer Receiving Chemotherapy. Cancer Epidemiol Biomarkers Prev.

[39] Lu J, Wang F, Ren Y, An Y, Ratti F, Marques HP (2025). Perioperative dynamic changes of systemic inflammatory biomarkers predict tumor recurrence following curative-intent resection of hepatocellular carcinoma. Eur J Surg Oncol.

