# Supplementary material for: Prognostic significance of dynamic changes in systemic inflammatory markers on mortality after liver transplantation: a retrospective cohort study
Source: Int J Med Sci. 2026 Jan 14;23(2):711–9. doi: 10.7150/ijms.126883 (PMC12825126; doi:10.7150/ijms.126883)
Supplement: Supplementary file 1 — Supplementary tables. [file ijmsv23p0711s1.pdf]

Table S1. Cause-specific mortality rate of recipients after liver transplantation

| Causes | |
|---|---|
| Infection | |
| Sepsis | 31 (43.1) |
| Pneumonia | 20 (27.8) |
| Cancer* | 9 (12.5) |
| Cardio- or Cerebrovascular events | |
| Cardiovascular | 2 (2.8) |
| Cerebrovascular | 4 (5.6) |
| Other† | 6 (8.3) |

Values are number of patient (%).

*, Progression of underlying hepatocellular carcinoma; †, Includes Hemorrhagic shock due to variceal bleeding, otherwise unknown

Table S2. Cox proportional hazard model of mortality after liver transplantation after excluding patient diagnosed opportunistic infection between surgery and postoperative NLR measurement

| | Univariate analysis | | | Multivariate analysis | | |
|---|---|---|---|---|---|---|
| | HR | 95% CI | *P*-value | HR | 95% CI | *P*-value |
| BMI, kg/m$^2$ | 0.88 | 0.82–0.94 | <0.001 | 0.90 | 0.84–0.97 | 0.005 |
| RRT | 1.83 | 0.93–3.57 | 0.079 | | | |
| MELD score | 1.03 | 1.01–1.05 | 0.005 | | | |
| deceased donor | 1.99 | 1.16–3.41 | 0.013 | | | |
| hemoglobin, g/dl | 0.88 | 0.78–0.98 | 0.022 | | | |
| platelet count, $10^3$/μl | 0.995 | 0.99–1.00 | 0.086 | 0.99 | 0.99–1.00 | 0.052 |
| C-reactive protein, mg/L | 1.01 | 1.00–1.02 | 0.040 | | | |
| Pre-and postoperative NLR-based group | | | | | | |
| Normal | | Ref | | | Ref | |
| Elevated | 0.82 | 0.11–6.06 | 0.842 | 0.71 | 0.10–5.31 | 0.738 |
| Normalized | 2.34 | 1.35–4.04 | 0.002 | 2.18 | 1.23–3.86 | 0.007 |
| Persistent | 6.25 | 3.13–12.52 | <0.001 | 4.96 | 2.34–10.51 | <0.001 |

NLR: neutrophil-to-lymphocyte ratio; HR: Hazard ratios; CI: Confidence intervals; BMI: body mass index; RRT: renal replacement therapy; MELD: Model for End-stage Liver Disease;

Table S3. Comparison between baseline demographic, clinical, and laboratory data between the Normal and Normalized groups

| | Normal group (n = 194) | Normalized group (n = 144) | *P*-value |
|---|---|---|---|
| Age, years | 58 (51, 63) | 56 (49, 63) | 0.073 |
| Sex, male | 134 (69.1) | 102 (70.8) | 0.727 |
| BMI, kg/m$^2$ | 24.0 (21.9, 26.9) | 23.8 (21.1, 25.5) | 0.108 |
| hypertension | 45 (23.2) | 39 (27.1) | 0.413 |
| diabetes mellitus | 71 (36.6) | 45 (31.3) | 0.306 |
| chronic kidney disease | 4 (2.1) | 14 (9.7) | 0.002 |
| RRT | 5 (2.6) | 29 (20.1) | <0.001 |
| MELD score | 10.4 (7.5, 15.8) | 21.6 (11.8, 34.7) | <0.001 |
| deceased donor | 53 (17.4) | 22 (30.6) | 0.012 |
| hemoglobin, g/dl | 11.2 (9.5, 12.8) | 9.2 (8.0, 10.9) | <0.001 |
| platelet count, 10$^3$/μl | 81 (57, 110) | 66 (46, 97) | 0.003 |
| glucose, mg/dl | 106 (91, 145) | 120 (95, 157) | 0.086 |
| creatinine, mg/dl | 0.72 (0.59, 0.91) | 0.96 (0.66, 1.68) | <0.001 |
| eGFR, ml/min/ 1.73m$^2$ | 106.0 (84.4, 130.5) | 72.7 (39.9, 115.9) | <0.001 |
| C-reactive protein, mg/l | 2.0 (0.8, 5.7) | 9.7 (3.1, 24.0) | <0.001 |
| alpha-fetoprotein, ng/dl | 4.3 (2.7, 10.1) | 4.9 (2.4, 13.0) | 0.883 |
| albumin, g/dl | 3.3 (2.8, 3.7) | 3.0 (2.7, 3.4) | 0.003 |

Values are number of patients (%) or median (interquartile range). BMI: body mass index; RRT: renal replacement therapy; MELD: Model for End-stage Liver Disease; eGFR, estimated glomerular filtration rate.

Table S4. Cox proportional hazard model of 3-year mortality after liver transplantation in the Normal and Normalized groups

| | Univariate analysis | | | Multivariate analysis | | |
|---|---|---|---|---|---|---|
| | HR | 95% CI | *P*-value | HR | 95% CI | *P*-value |
| chronic kidney disease | 1.57 | 0.57–4.34 | 0.387 | | | |
| RRT | 1.94 | 0.95–3.96 | 0.070 | | | |
| MELD score | 1.04 | 1.02–1.06 | <0.001 | 1.02 | 1.00–1.05 | 0.08 |
| deceased donor | 2.27 | 1.27–4.04 | 0.005 | | | |
| hemoglobin, g/dl | 0.86 | 0.76–0.98 | 0.022 | | | |
| platelet count, $10^3$/μl | 0.997 | 0.99–1.00 | 0.334 | | | |
| creatinine, mg/dl | 1.12 | 0.90–1.40 | 0.323 | | | |
| eGFR, ml/min/ 1.73$m^2$ | 0.996 | 0.99–1.00 | 0.123 | | | |
| albumin, g/dl | 0.89 | 0.55–1.42 | 0.614 | | | |
| C-reactive protein, mg/l | 1.01 | 1.00–1.02 | 0.053 | | | |
| Pre-and postoperative NLR-based group | | | | | | |
| Normal | | Ref | | | Ref | |
| Normalized | 2.38 | 1.38–4.11 | 0.002 | 1.93 | 1.03–3.62 | 0.040 |

HR: Hazard ratios; CI: Confidence intervals; RRT: renal replacement therapy; MELD: Model for End-stage Liver Disease; eGFR: estimated glomerular filtration rate; NLR: neutrophil-to-lymphocyte ratio.
